# Differentiation of pluripotent stem cells for modeling human skin development and potential applications

**DOI:** 10.3389/fcell.2022.1030339

**Published:** 2022-11-23

**Authors:** Fabian Oceguera-Yanez, Alfonso Avila-Robinson, Knut Woltjen

**Affiliations:** ^1^ Department of Life Science Frontiers, Center for iPS Cell Research and Application (CiRA), Kyoto University, Kyoto, Japan; ^2^ Tecnologico de Monterrey, Campus Santa Fe, Mexico City, Mexico

**Keywords:** human induced pluripotent stem cells, iPSCs, epidermal differentiation, 3D skin models, skin organoids, skin inherited diseases, keratinocyte

## Abstract

The skin of mammals is a multilayered and multicellular tissue that forms an environmental barrier with key functions in protection, regulation, and sensation. While animal models have long served to study the basic functions of the skin *in vivo*, new insights are expected from *in vitro* models of human skin development. Human pluripotent stem cells (PSCs) have proven to be invaluable tools for studying human development *in vitro*. To understand the mechanisms regulating human skin homeostasis and injury repair at the molecular level, recent efforts aim to differentiate PSCs towards skin epidermal keratinocytes, dermal fibroblasts, and skin appendages such as hair follicles and sebaceous glands. Here, we present an overview of the literature describing strategies for human PSC differentiation towards the components of skin, with a particular focus on keratinocytes. We highlight fundamental advances in the field employing patient-derived human induced PSCs (iPSCs) and skin organoid generation. Importantly, PSCs allow researchers to model inherited skin diseases in the search for potential treatments. Skin differentiation from human PSCs holds the potential to clarify human skin biology.

## 1 Introduction

The discovery of induced pluripotent stem cells (iPSCs) by Yamanaka and others (Takahashi and Yamanaka, 2006; Takahashi et al., 2007) provided new strategies to understand skin morphogenesis. Human iPSCs are akin to embryonic stem cells (ESCs) in their unlimited proliferation capacity and ability to differentiate into the three different embryonic lineages - endoderm, mesoderm, and ectoderm - the latter two being precursors of dermis and epidermis of the skin, respectively. It is expected that human pluripotent stem cells (PSCs) will be able to recapitulate the complexity of human skin (Khurana et al., 2021). Research on skin reconstructed from PSCs can be translated to develop clinical strategies that may alleviate skin inherited diseases. Hence it is important to gather the knowledge to develop scalable differentiation protocols into relevant skin cells. Here, we will discuss key issues in the differentiation of human PSCs into keratinocytes and the derivation of disease-specific or patient-derived human iPSCs and skin organoid generation.

## 2 Basic biology of the skin

**FIGURE 1:**
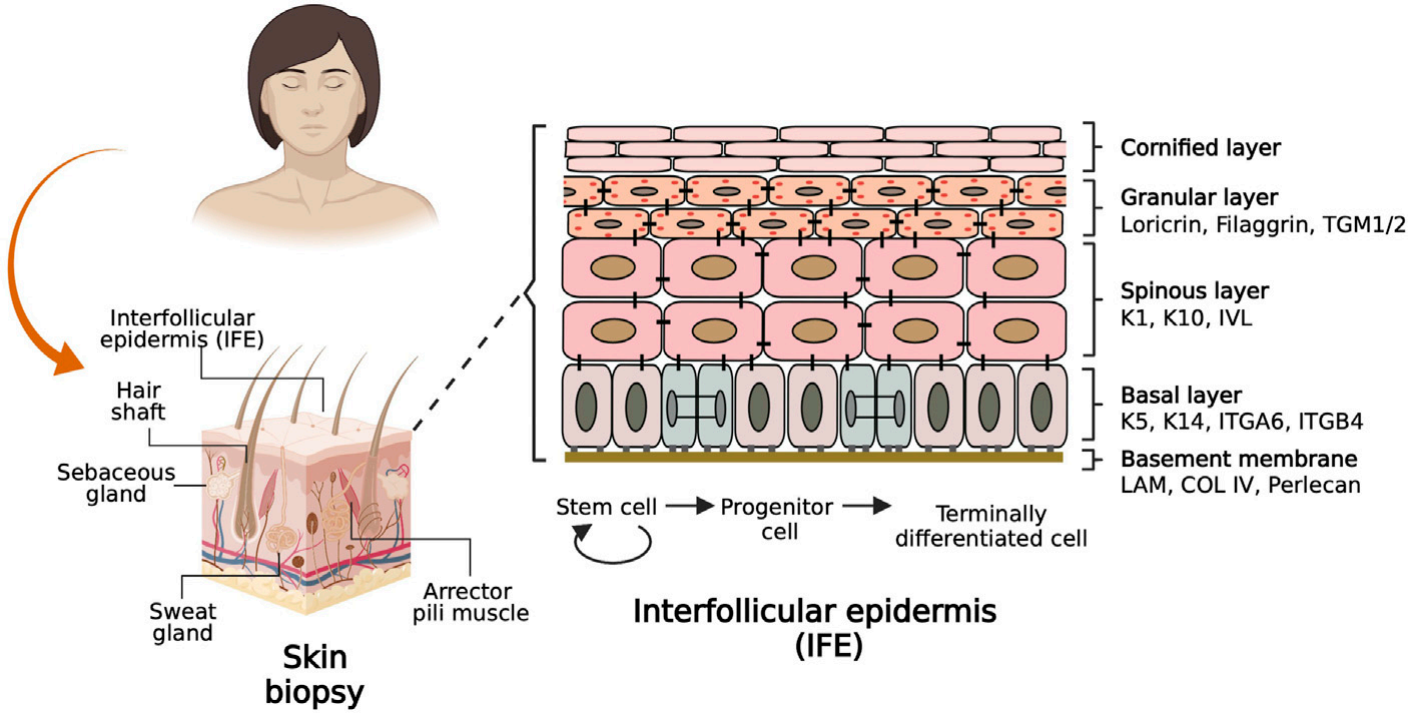
Basic biology of the skin. The scheme on the left shows a sagittal section of a skin biopsy depicting the main components of the skin labelled with associated skin appendages. The scheme on the right shows a basement membrane adjacent to the interfollicular epidermis (IFE) populated by epidermal stem cells keratinocytes that differentiate to reconstitute four histologically distinct layers. The keratinocyte molecular markers characteristic of each layer are labelled on the side.

The skin epidermis is an organ conformed by the interfollicular epidermis (IFE) and associated appendages such as hair follicles, sweat glands, and sebaceous glands (Figure 1), which provide sensory perception and lubrication, among other important physiologic functions (Fujiwara et al., 2018). The cellular entity of the skin epidermis is the keratinocyte, which possesses cell plasticity that allows it to regenerate and recreate the tissue during homeostasis and upon injury. The skin epidermis is maintained by epidermal stem cells located at the basal layer and in the vicinity of skin appendages. Epidermal stem cells undergo cell division to self-renew and generate fast-dividing keratinocyte progenitor cells that repopulate the tissue during homeostasis and upon injury (Solanas and Benitah, 2013).

The epidermis is composed of four histologically distinct layers (Figure 1, right). The basal layer contains stem cells and keratinocyte progenitor cells that can be identified by the expression of Keratin 5 and Keratin 14 (K5/K14) heterodimers. As keratinocytes from the basal layer commit to differentiate terminally, they migrate to the suprabasal or spinous layer, whose cells are densely interconnected by desmosomes and are characterized by expression of K1/K10 and Involucrin. Immediately above this layer lies the granular layer, which expresses Filaggrin and Loricrin proteins which are precursors of the skin barrier. Furthermost above resides the cornified layer composed of anucleated cells that form a lipid barrier layer which provides the mechanical defense character of the skin. Juxtaposed to the basal layer of the epidermis resides the dermis, a layer of connective tissue composed of fibroblasts, blood vessels, nerves, and immune cells. Dermis and epidermis are separated by a basement membrane, which consists mainly of four subtypes of extracellular matrix (ECM) proteins: Laminin, Collagen type IV, Nidogen, and Perlecan, which are deposited by epidermal and dermal cells and serve both as an anchoring structure to the basal epidermal cells while also providing signaling cues that determine cell fate and function (Chen and Gumbiner, 2006; Watt and Fujiwara, 2011; Breitkreutz et al., 2013).

The vertebrate skin epidermis originates from the embryonic surface ectoderm, which is a single layer of ectodermal cells covering the embryo after neurulation. The surface ectoderm consists of flat cells that form a simple epithelium characterized by the expression of K8/K18 heterodimer (Moll et al., 1982 and reviewed in Liu et al., 2013; Koster and Roop, 2007). The expression of the transcription factor (TF) TP63 is an early event during skin development. TP63 regulates the proliferation of epithelial stem cells and is essential for skin differentiation. TP63 knockout mice showed abnormalities in epidermis development and stratification resulting in neonatal death, possibly due to dehydration (Mills et al., 1999; Yang et al., 1999). Two alternative promoters regulate the expression of TP63 isoforms. TA-p63 contains an N-terminal transactivation domain (TA) with high homology to TP53, while the dN-p63 isoform lacks the TA domain (Yang et al., 1998). Both TP63 isoforms are expressed in the developing epidermis and positively influence the expression of K14. TA-p63 mediates K14 promoter transactivation by TFAP-2γ, and dN-p63 binds an enhancer in the K14 promoter to activate expression (Byrne et al., 1994; Koster and Roop, 2007). The expression of K5/K14 heterodimer coincides with the decline of K8/K18 expression, an event that hallmarks the commitment to epidermal fate (Liu et al., 2013).

## 3 Differentiation of human pluripotent stem cells into dermal and epidermal lineages

In the following sections, we will discuss three main approaches to differentiate human pluripotent stem cells (PSCs) into epidermal lineages (Table 1). First, we summarize *in vitro* differentiation protocols using human iPSCs or ESCs with functional outcomes. The second section reports differentiation protocols using direct conversion. The third section details differentiation protocols leading to 3D-skin organoids. In all cases, variations in the ECM utilized, supplementing compounds, and the medium employed are included in Table 1. A full list of differentiation protocols is provided in Supplementary Table S1.

**TABLE 1:** Differentiation protocols using PSCs.

Originating cells	Medium composition	Growth factors and inhibitors	ECM	Proteins expressed	Functional outcome	Reference (s)	
A) *In vitro* differentiation							
hiPSCs	D-KSFM => KSFM	RA, BMP4	Matrigel=> fibronectin, collagen	p63, K14, DSG3, K1, COL7A1, Laminin 5, Loricrin	3D skin equivalents	(Itoh et al., 2011; Kajiwara et al., 2017; Matsumura et al., 2018)	
hESCs (H9, H13, H14); hiPSCs	D-KSFM	SU6656 (SFK: Src Family Kinase inhibitor); RA, BMP4	Matrigel	K8, K18, K14, K3, K10, IVL	Calcium increase led to IVL and K10 expression	Lian et al. (2013)	
hESCs (H1)	a) FAD => D-KSFM; b) D-KSFM	a) Autologous feeder, AA, RA (20days) => Activin; b) Decellularized feeders, RA	a) Autologous ESC-derived Fbs, Col IV; b) Decellularized autologous ESC-derived Fbs+Ficoll, Col IV	p63, K14	N.D.	Movahednia et al. (2015)	
h 209.2 PSC	D-KSFM; CNT-07 after D4	RA, BMP4 (until D4); ROCKi [Y-27632] (after D10)	Fibronectin, Col IV	p63, INTα6, INTβ4, COL17A1; C1ORF68, LCE2B, TGM1, TGM2	ESPCs exhibited basal cell phenotype	Ruiz-Torres et al. (2021)	
hiPSCs	FAD D4, N2 D7; D-KSFM	RA+ROCKi D2; RA+BMP4 D7	Gelatin => MEF=> CELLstar	K18, p63, K14	3D skin equivalents; Mouse skin xenografts	Sebastiano et al. (2014)	
hESCs (H1)	DMEM/F12 (1:1) until D10; Thereafter D-KSFM for KC differentiation	BMP4, FCS+/-DAPT	Matrigel, Col I	K18, p63, K14	Immunostaining of monolayer cultures	(Tadeu and Horsley, 2013; Tadeu et al., 2015)	
hiPSCs	1. FAD: DMEM/F12 (3:1); 2. D-KSFM; 3. KSFM	Initially low BMP4, followed by RA, then RA + BMP4+EGF, then BMP4+EGF, finally EGF alone	Matrigel	CD200, ITGA6, K15, K5, K14, ITGB1, K1, K10	3D-skin equivalents; HF patch assay;	Yang, et al. (2014)	
			Differentiation on 3T3 cells		Skin Reconstitution assay. Permeability assays (toluidine blue dye)		
B) Direct conversion							
Human fibroblasts (Fbs)	KSFM	Retroviral transduction of p63 and KLF4	-	K14, GJB2	Induced KCs expressed KC-specific proteins/KC-similar transcriptome	Chen et al. (2014)	
		EGF, bovine pituitary extract					
Dermal Fbs and adipose-derived stromal cells		Lentiviral transduction of dN-p63A, GRHL2, TFAP2-A and cMYC		K14, K13, K10	Transplantation into skin wounds in mice	Kurita et al. (2018)	
Human Fbs		Oct4, Sox2, Klf4, and c-Myc		K14, KRT19, ITGB1 and K1	Calcium increase led to K1 expression	Zhao et al. (2020)	
		RA, BMP4, hEGF and bFGF					
C) 3D-skin organoids							
hiPSCs	D-KSFM + Suppl;	RA, BMP4;	-	K1, K10, K14, VIM	Floating skin organoids (FSO), xenotransplantation of differentiated in wounded mice	(Ebner-Peking, 2021)	
	Endothelial Basal Medium + Suppl. (EGM)						
		FGF-7 mRNA					
hiPSCs	EB: DMEM:F12 (D0-8);	RA, BMP4, EGF;	Col IV (KCs); Col I (organoid)	dN-p63, K5, K14, Loricrin	3D skin organoid, engraftment in mice	Kim and Ju, (2019)	
	KCs: D-KFSM (D9-12);						
		BMP4, EGF					
	D-KFSM:KSFM (D13-30)						

### 3.1 *In vitr*o differentiation

#### 3.1.1 Morphogen-mediated induction of early skin

Studies of embryonic skin development using various animal models have helped identify appropriate culture conditions for PSC differentiation along the necessary lineages to recreate the layers of the skin. Retinoic acid (RA) fulfills many important functions during the development of epidermis, the limbs, and the secondary palate (Mammadova et al., 2016). Impaired skin differentiation and abnormal stratification were observed in mice overexpressing a dominant-negative form of the retinoic acid receptor (RAR) under the regulation of the Keratin 14 promoter, capable of inhibiting the endogenous activities of RAR α, β, and γ (Saitou et al., 1995). Andrews reported that treating human ESCs with RA was sufficient to induce differentiation into neural ectoderm lineages (Andrews, 1984). Palecek and others applied RA to differentiate human ESCs into keratinocytes resulting in a significant increase in the number of cells expressing dN-p63 and KRT14, followed by expression of the terminal epidermal differentiation markers KRT10, Involucrin and Filaggrin (Metallo et al., 2008). Furthermore, topical application of RA to human epidermal keratinocytes cultured *in vitro* sustained expression of the dN-p63a isoform and stimulated proliferation of skin progenitor cells (Bamberger et al., 2002).

*In vivo*, BMP4 works both as a potent inducer of the epidermis and also as a neuralization inhibitor (Wilson and Hemmati-Brivanlou, 1995). It has been shown that BMP4 induces transactivation of the dN-p63 promoter in Zebrafish and *Xenopus*, and positively balances fate commitment from neural into non-neural ectoderm (Bakkers et al., 2002; Tribulo et al., 2012). BMP4 treatment induces apoptosis in mouse Sox-1+ve neural ectoderm progenitors, thus favoring the development of epidermal lineages and limb appendages (Gambaro et al., 2006). Therefore, BMP4 determines commitment of the epidermis during embryonic development (Itoh et al., 2011; Kajiwara et al., 2017; Larribere et al., 2017).

**FIGURE 2:**
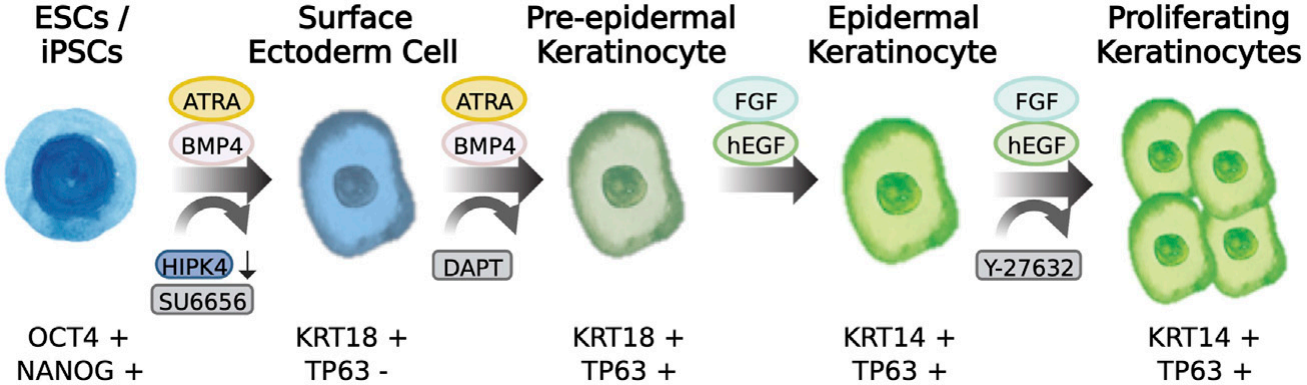
Differentiation of pluripotent stem cells to skin keratinocytes. Scheme showing the transition from a pluripotency state to epidermal keratinocytes incorporating morphogen-induction and compound induction of skin epidermis. The cell markers characteristic of the cell state is described below the drawings.

WNT is an important morphogen controlling different events of development and stem cell behavior. WNT elicits decisive signals on the ectoderm layer to define whether neural or epidermal lineage will form (Wilson et al., 2001; Lim and Nusse, 2013). WNT signals prevent the inhibitory actions of FGF on BMP expression, and thereby favor the commitment of epidermis while opposing formation of neural lineages. Notwithstanding the important roles for WNT signaling in the generation of epidermis, there are few reports describing the use or application of WNT agonists in the differentiation of PSCs to epidermal cells. Chen and others described in their protocol that addition of CHIR99021, a WNT pathway activator, could work together with BMP4 stimulation and TGFβ inhibition to induce robust TP63 expression and at the same time decreased the expression of PAX6. Thus, WNT activation balances the determination of the epidermal lineages while suppressing the neural fate (Zhong et al., 2020), The aforementioned reports pioneered the development of keratinocyte differentiation protocols from PSCs. More recently, through the application of various techniques such as RNA-seq, single-cell profiling, and automated light microscopy allowed to study early skin development and enabled manipulation of the signaling pathways involved in the lineage determination from pluripotency to surface ectoderm and ultimately commitment to skin keratinocytes (Figure 2).

#### 3.1.2 Modulation of skin development

Based on the important role of TP63 during epidermis development, studies of dominant-negative TP63 isoforms should help reveal key events occurring during skin morphogenesis. Fibroblasts obtained from patients suffering from ectrodactyly, ectodermal dysplasia and cleft lip/palate (EEC) syndrome caused by somatic mutations in the DNA-binding domain of TP63, were reprogrammed to iPSCs (Soares et al., 2019). It was observed that EEC syndrome iPSCs were incapable to differentiating to epithelial or epidermal lineages when induced *in vitro*. Bulk and single-cell RNA seq analyses were performed in order to profile the differences during epidermis differentiation between normal and EEC patient iPSCs. The study identified sets of genes regulated by TP63 during epithelial commitment. EEC-iPSCs showed elevated expression of mesodermal genes such as vimentin (VIM) and matrix metalloproteinase family (MMP) and were correlated with non-epithelial cells such as skeletal muscle myoblasts, lung fibroblasts, or umbilical vein endothelial cells. The above observations strongly suggested that TP63 activates epithelial and epidermal genes and possibly represses mesodermal genes during skin development. The results of the study informed the authors to apply compounds known to repress mesoderm induction, such as heparin, suramin, and valproic acid (VA). Heparin can bind and sequester FGFs, suramin is a potent inhibitor of VEGF and FGFs, and VA is a histone deacetylase inhibitor that up-regulates H3 acetylation and represses EMT. When the inhibitors were included in the differentiation medium of EEC patient iPSCs, epithelial differentiation was restored enabling the expression of the epidermal genes KRT5, EDAR2, DSG3, and LAMA3 (Soares et al., 2019).

Horsley and others performed experiments using transgenic mice and human ESC differentiation in parallel to analyze the early stages of surface ectoderm commitment towards keratinocytes and identified Notch signaling activity prior to TP63 expression (Tadeu and Horsley, 2013). Following application of DAPT, a γ-secretase inhibitor that blocks Notch signaling, the expression of TP63 increased during the initial stages of surface ectoderm determination of both human ESCs and mouse embryonic skin (Tadeu and Horsley, 2013). In a follow-up study, RNA-sequence analysis characterized the transcriptional profiles of human ESCs differentiated with BMP4 under γ-secretase inhibition. Treatment of human ESCs with DAPT induced an early adoption of surface ectoderm fate caused by expression of a set of transcription factors for development of epidermis including IRF6, important for normal skin, limb, and craniofacial morphogenesis and TP63, DLX3, BARX2, EDAR, and SFN which regulate epidermal keratinocyte fate induction (Tadeu et al., 2015). Epidermis-mesenchymal interactions were also identified in the analysis, such as LMX1, whose expression induces a dorsal epidermal fate in the vertebrate limb, and PTN, which is a mesenchymal regulator of epithelial embryonic development (Tadeu et al., 2015).

Various groups have converged on a common pitfall while performing epidermis differentiation experiments using PSCs, namely decreased proliferation capacity following differentiation (Itoh et al., 2011; Kidwai et al., 2013; Sebastiano et al., 2014 and reviewed in Rheinwald, 2013 and Khurana et al., 2021). Indeed, it was suggested that cell expansion may be limited by telomere length shortening and cell senescence mechanisms (Kidwai et al., 2013). For example, p16 INK4A activates and imposes a break in keratinocyte proliferation that can be alleviated by overexpression of BMI1, a repressor of p16 INK4A, and p14 ARF signals (Movahednia et al., 2015). Recently, Sah et al. (2021) reported the generation of single iPSCs into homogenously differentiated keratinocytes through the addition of RA and BMP4, thus avoiding the generation of EBs or colony passaging. In order to maintain the proliferative capacity of their human iPSC-derived keratinocytes, they included the ROCK inhibitor Y-27632 in their culture protocol and were able to achieve moderate passaging of their induced keratinocytes nine times. After increasing the Ca2+ concentration to >1.2 mM in the medium, the keratinocytes expressed terminal differentiation markers KRT1, Filaggrin, and Loricrin. Ruiz-Torres et al. (2021) reported the use of a similar protocol to derive epidermal TP63 positive (+ve) cells that expressed ITGA6, ITGB4, and COL17A1 within 20 days with the capacity to be passaged four times. The resulting cells were capable of forming stratified epidermis that express cornified layer markers TGM1 and TGM2. The development of systematic studies to identify and overcome proliferation arrest mechanisms may allow the identification of alternative molecules that improve the proliferation capacity of PSC-derived keratinocytes.

β-catenin is a key molecule of Wnt signaling, serving a pivotal role by changing localization between the nucleus and membrane. When localized at the membrane, β-catenin interacts with E-cadherin and controls the structure and mechano-transduction of adherens junctions. When localized in the nucleus, β-catenin interacts with transcription factors and transactivates the expression of WNT/β-catenin responsive genes (Valenta et al., 2012). Tyrosine kinases can also control the spatio-temporal localization of β-catenin and its interaction with E-cadherin through phosphorylating β-catenin at Y654 residue. Application of the Src kinase inhibitor (SU6656) to pluripotent cells increased membrane-bound β-catenin, resulting in epithelial cells expressing KRT8 and KRT18 (Lian et al., 2013), while OCT3/4 expression was downregulated. Interestingly, the epithelial cells were able to undergo surface ectoderm differentiation upon treatment with RA and BMP4 in the differentiation media, generating either KRT14 epidermal or KRT3 corneal epithelial progenitors. Importantly, the progenitor cells could be expanded in culture to about 24 population doublings (Lian et al., 2013; Selekman et al., 2016). Although this process can generate multipotent epithelial progenitor cells, it enables the production of a great number of cells that could later commit to epidermal keratinocyte lineage.

A microscopy-based screen combined with RNA interference investigated the roles of kinases and kinase-regulatory proteins in early skin epithelial differentiation (Larribere et al., 2017). The screen was designed to identify molecules that influence the generation of KRT18 + ve cells and thereby improve epithelial progenitor cell generation. They identified 35 previously reported genes which function in epidermis differentiation, including BMP receptors, IKK-related genes, and MAPKs, validating the study (Larribere et al., 2017). HIPK4, a homeodomain interacting protein kinase 4, was identified as a crucial inhibitor of KRT18 expression. HIPK4 expression was detected in the developing skin of E17.5 mouse embryos. Down-regulation of HIPK4 using RNAi was found to substantially increase the number of KRT18 + ve cells from 50% to 85% after differentiation induction, although KRT14 +ve cells increased only moderately from 50% to 65%. Transcriptome profiling of HIPK4 knockdown samples identified IL-6 and TGFβ/BMP as signaling pathways that could be increased in a HIPK4-dependent manner (Larribere et al., 2017). Similar experimental efforts may lead to better understand the signals required for epidermal morphogenesis and will likely aid in the improvement of differentiation protocols in the future.

#### 3.1.3 Differentiation into dermal fibroblasts

Proper formation of human skin requires the mutual interaction of epidermal and dermal cells. An elaborate protocol to derive dermal fibroblasts was described (Nakajima et al., 2018; Nakajima et al., 2019). The authors followed the rational provided by developmental cues identified in chick and mouse embryos undergoing somitogenesis. Induction of somitic mesoderm was initiated by the addition of a combination of chemical compounds including an inhibitor of TGFβ (SB431542), a GSK3β inhibitor to mimic WNT activation (CHIR99021), and a BMP antagonist (DMH1). Dermomyotome cells were identified by expression of Engrailed 1 (EN1) and PDGFRa receptor. Subsequent addition of BMP4 and CHIR99021 enabled the generation of dermal fibroblasts. The resulting cells expressed Collagen I and Hyaluronic acid. Alternative attempts at reconstituting skin from PSCs involved layering fibroblasts and keratinocytes differentiated from iPSCs (Kim et al., 2018). Embryoid bodies prepared from iPSCs were deposited on matrigel, and migrating cells were treated with BMP4. Selective attachment to non-coated plastic followed by Collagen I adhesion led to cells with fibroid morphology. The resulting fibroblasts expressed CD105, CD73, and CD44, and were capable of secreting the extracellular matrix proteins fibronectin and vimentin. iPSC-derived fibroblasts were immersed in Collagen I gels and cultured on tissue culture inserts overlayed with iPSC-keratinocytes and later exposed to the air-liquid interface to allow reconstitute the different layers of epidermis *in vitro*. Transplanting the skin generated *in vitro* onto immunodeficient mice resulted in the expression of mature markers K14, Loricrin and Involucrin, functionally validating the assay.

Christiano and others reported an alternate protocol to derive fibroblasts from iPSCs. By allowing iPSCs to form embryoid bodies in medium including TGFβ2 and ascorbic acid, fibroblast-like cells developed (Itoh et al., 2013). The majority of differentiated fibroblasts expressed the dermal fibroblast markers CD10, CD44, CD73 and CD90. These iPSC-fibroblasts were functionally tested by grafting onto the back of SCID mice in combination with normal human keratinocytes, and resulted in reconstitution of skin expressing Keratin 1, Loricrin and Collagen VII at the basement mebrane. Ascorbic acid has been reported to induce mesodermal lineages from PSCs and also to enhance Collagen synthesis, an important measure of ECM deposition by dermal fibroblasts (Shamis et al., 2012), Importantly, the PSC-derived fibroblasts that developed were able to support the growth and stratification of 3D-reconstituted skin when co-cultured with iPSC-keratinocytes (Itoh et al., 2013; Kim et al., 2018).

### 3.2 Direct conversion and directed differentiation

Direct conversion is a process whereby cells undergo cell-fate changes from their current developmental lineage onto a different and unrelated somatic state. Direct conversion may be facilitated by overexpression of a lineage-defined cocktail of transcription factors (TFs), such as Ascl1, Pou3f2 and Myt1l for converting mouse fibroblasts into functional neurons *in vitro* (Vierbuchen and Wernig, 2011). Conversion of human fibroblasts to keratinocytes was achieved by the overexpression of two TFs, TP63 and KLF4 (Chen et al., 2014). The resulting keratinocytes expressed KRT5/KRT14 and global gene expression profiling confirmed similarities with epidermal keratinocytes. TFs may also be used to direct differentiation of PSCs into a variety of somatic lineages including skeletal muscle by the overexpression of MyoD (Tanaka et al., 2013), or cortical neurons by overexpression of Ngn2 (Kondo et al., 2017), or combined expression of Ascl1, Pou3f2 and Myt1l and NeuroD1 (Pang et al., 2011). Direct conversion of mouse fibroblasts into functional keratinocytes has been described (Iacovides et al., 2016). In their approach, transient expression of pluripotency-associated TFs Sox2, Oct4 and Klf4 initiated transcriptional reprograming towards pluripotency and was followed by the addition of RA and BMP4 into keratinocyte medium to induce MEF conversion along an ectodermal fate (Iacovides et al., 2016). Conversion of human fibroblasts to keratinocyte stem-like cells was also reported *via* brief overexpression of the reprogramming factors Oct4, Sox2, Klf4, and c-Myc using retroviruses (Zhao et al., 2020), followed by the addition of RA, BMP4, hEGF, and bFGF into keratinocyte medium. The resulting keratinocytes expressed ITGβ1 and KRT19, and upon increasing the extracellular Ca2+ concentration the cells upregulated KRT1 and downregulated KRT14 (Zhao et al., 2020). Since KRT19 is associated with periderm development, it is possible that the approach generated early epidermal lineages (Moll et al., 1982). More recently, it was reported that decreasing the expression of KLF4 using siRNA resulted in retardation of the terminal differentiation program with augmented proliferation of immature keratinocytes derived from ESCs (Fortunel et al., 2019). The function of Klf4 in mouse skin was initially ascribed as a transcription factor involved in the establishment of the cornified layer of the epidermis, as Klf4 was found to repress the expression of proteins involved in the cornified envelope formation-related protein Sprr2 (Segre et al., 1999). Thus, Klf4 can exert different functions during skin epidermis development, suggesting future applications to improve PSC differentiation to epidermis.

Kurita et al. (2018) reported the reprograming of dermal fibroblasts and adipose-derived stromal cells into keratinocytes with the aim of restoring wound closure for the treatment of large cutaneous ulcers. Amongst 55 TFs, four key TFs, dN-p63A, GRHL2, TFAP2-A and cMYC (DGTM), were identified and delivered by lentiviral particles into the mesenchymal cells resident in wounds generated on the back of mice. When mice were wounded to generate artificial ulcers and treated with the DGTM cocktail, the mesenchymal cells changed onto a keratinocyte lineage expressing Keratin 14. The newly formed epithelial cells closed the wound and stratified to form a suprabasal layer of cells expressing Keratin 13, which progressively switched to express the adult epidermal Keratin 10. The barrier permeability test based on Toluidine blue dye incorporation showed that the generated epidermis excluded the dye from the inner tissue layers and therefore compares functionally with that of skin epidermis formed with primary keratinocytes (Kurita et al., 2018). This work has a great potential to contribute to the medical field in the treatment of chronic ulcers, thermal burns and surgical incisions, amongst many other applications. Alternative skin resident cell lineages such as melanocytes have also been induced by overexpression of three transcription factors into human fibroblast. The TFs SOX10, MITF and PAX3 were identified after screening a pool of candidate transcription factors related to neural crest lineage determination and were sufficient to reprogram human fibroblasts to functional melanocytes (iMels). The resulting melanocytes are capable of generating melanosomes and transferring melanin granules into the surrounding keratinocytes in 3D-reconstituted skin and skin reconstitution assays in mice *in vivo* (Yang et al., 2014).

### 3.3 3D-skin organoids

Organogenesis of skin appendages occurs early in embryonic development through a series of reciprocal interactions between the dermis and epidermis, and the roles of FGFs, TGFβs, sonic hedgehog (Shh) and BMP signaling molecules in these processes has been established previously (Kishimoto et al., 1999). Therefore, reconstituting these two main components of the skin is expected to improve organogenesis of skin appendages. Tsuji and others performed a variety of experiments to reconstitute the skin and associated appendages. Embryoid bodies (EBs) generated with mouse iPSCs, immersed within a collagen gel were grafted as clusters into the subrenal capsule of severe combined immunodeficient (SCID) mice (Takagi et al., 2016). Gastric, respiratory and skin epithelium could be generated. Interestingly, treatment of EBs with WNT10b increased the number and maturity of hair follicles and associated structures such as dermal papillae and sebaceous glands. The subsequent orthotopic transplant of the skin organ system generated into the back skin of nude mice allowed the engraftment of bioengineered hair follicles and establishment of connections with arrector pili muscles within the recipient mice, highlighting important functional properties. Recently, skin organoid generation was reported using aggregates of human iPSCs (Lee et al., 2020). Treatment of iPSC EBs with BMP4 to induce surface ectoderm gave rise to epidermis, followed by a morphogen cocktail containing FGF2 to induce cranial neural crest cells that eventually converted into dermis. After allowing the aggregates to mature in culture for 70 days, a variety of appendages were identified that included hair follicles pigmented by melanocytes, innervating neurons, and sebaceous glands. Because the neural crest develops into cranial dermis, the described hair follicles resembled facial beard and ear hair. The work provided proof of concept of the potential of iPSCs to regenerate skin appendages, and underscores the capability of such systems to test adequate morphogens in an orderly manner to derive skin from different locations in the human body (Lee et al., 2020; Sun et al., 2021; Lee et al., 2022). Ebner-Peking et al. (2021) reported the assembly of skin spheroid organoids containing vasculature by culturing together human iPSCs-derived keratinocytes, fibroblasts, and endothelial cells. Once assembled, the completely iPSC-derived skin organoids were grafted into immune-deficient mice. Three months later, the histology showed a clear epidermal and dermal organization containing Ki67+ve proliferating hiPSC-derived epidermal cells. The dermis developed from hiPSCs showed an intricate vasculature identified by human-specific anti-CD31 antibodies in immunostaining experiments (Ebner-Peking et al., 2021).

Despite the rapid advances in the development of skin organoids, they still lack crucial features for expanding their use. As described by Lee and Koehler (2021), a shared pitfall of the current organoid models reside in the absence of relevant cell populations, such as endothelial cells, pericytes, and immune cells (eg., Langerhans cells and T cells); skin organoids are deficient of vasculature and sweat glands development, thus opening new avenues for research.

## 4 Disease modeling

Inherited disorders of the skin are generally caused by mutations in the genes encoding for intermediate filaments and structural proteins such as keratins or collagens. They span a diverse group of blistering and disfiguring diseases and affect many individuals in every ethnic group (Morren et al., 2022). The use of iPSCs for the development of personalized medicine therapies holds great promise in the treatment of diseases caused by genetic disorders (Khurana et al., 2021), and a clear understanding of the pathways affected will help to develop therapeutic cures. The dermatology field could benefit from making use of banks of iPSCs already generated from different diseases (Seltmann et al., 2016; De Sousa et al., 2017). In Supplementary Table S2 we present examples of iPSC-derived skin disease models and highlight the cell lineages differentiated to reach the research outcome.

Recently, iPSCs-derived skin organoids have been applied as novel tools for disease modeling and drug screening. Ramovs et al. (2022) reported the characterization of hair-bearing skin organoids by reproducing the protocol of Lee et al. (2020) and carefully examining the epidermal-dermal junction to evaluate the suitability of skin organoids to model diseases such as epidermolysis bullosa (EB). The skin organoids displayed a fully stratified interfollicular epidermis, however, the epidermal-dermal junctions reconstituted were almost devoid of Collagen VII, indicating their incomplete maturation; a potential complication for EB modeling. Ma et al. (2022) developed iPSC-derived epidermal and mesenchymal-like organoids following the protocol of Lee et al. (2020) and grafted them into a localized scleroderma mouse model with the goal of alleviating disease symptoms. Localized scleroderma presents as a connective tissue disease represented by fibrosis and abnormalities in the immune system and blood vessels and can be induced in mice by Bleomycin injection. It was reported that scleroderma mice grafted with skin organoids showed a decrease in the levels of inflammatory cytokines, a reduced accumulation of abnormal ECM deposition accordant with an increase in blood vessel development (Ma et al., 2022). Thus, the application of skin organoids into localized scleroderma may help to discern therapeutic strategies.

## 5 Future perspectives

Despite the diversity of keratinocyte differentiation protocols, most converge on the manipulation of a handful of signaling pathways controlling early skin morphogenesis. Beyond this, we propose alternative strategies to be implemented for the improvement of *in vitro* keratinocyte differentiation from human PSCs.

Human iPSCs from different sources demonstrate variability in differentiation efficiency (Osafune et al., 2008; Hu et al., 2010; Bock et al., 2011). While optimization of morphogen or small molecule concentrations may resolve these differences, it is recommended that different PSCs be tested for their differentiation propensity towards the desired lineage. Epigenetic memory may play an important role (Kim et al., 2011). The intrinsic capability of keratinocyte-derived iPSCs to generate keratinocytes was applied advantageously, although the impairment in proliferation of the pluripotent-epidermal derivatives still remained an important issue (Sebastiano et al., 2014; Matsumura et al., 2018). Naïve PSCs show markedly reduced H3K27me3 transcriptional silencing histone marks compared to primed PSCs (Ware, 2017). It is therefore hypothesized that naïve PSCs may have improved potential to differentiate into any specific developmental lineage due to resetting of their epigenetic status.

Recent technical advances in single cell sequencing and analyses of nuclear chromatin organization have offered clarity on the succession of events underlying epidermis and dermis communication (Fan et al., 2018). We expect that further studies will uncover the processes of human skin morphogenesis, which in turn could be applied to broadly improve differentiation protocols.

The generation of reporter PSC lines that enable quantitative comparisons of differentiation capacity can aid in comparisons of various differentiation protocols. A relevant example is provided by the generation of a TP63 knock-in human iPSC line that permitted the identification of cell lineages expressing TP63 during corneal epithelial development (Kobayashi et al., 2017). Similar approaches to generate knock-in fluorescent reporters for epidermal genes will prove beneficial for the comparison and standardization of protocols to generate iPSC-derived epidermal keratinocytes.

## 6 Conclusion

Our understanding of the fundamental mechanisms regulating PSC differentiation towards keratinocytes and skin appendages has expanded in the last decade, driven by advances in iPSC technologies and developmental biology. As evidenced by several reports reviewed herein, PSC research has led to the development of a broad toolkit of differentiation methods capable of generating functional keratinocytes, fibroblasts, and even the formation of complex skin organoids. As further refinement of PSC differentiation strategies will open a path to regenerative medicine for skin wounds and disease, challenging current barriers is necessary and warranted in the field.
