# Supplementary material for: Differentiation of pluripotent stem cells for modeling human skin development and potential applications
Source: Front Cell Dev Biol. 2022 Nov 23;10:1030339. doi: 10.3389/fcell.2022.1030339 (PMC9728031; doi:10.3389/fcell.2022.1030339)
Supplement: Supplementary file 1 [file Table1.docx]

**Supplementary Information 1**

Complete list of keratinocyte differentiation protocols using human iPSCs or ESCs with functional outcomes reported in the experiment and variations in the ECM utilized, supplementing compounds, and the medium employed.

| **Cell type** | **Medium composition** | **Growth factors & compounds** | **ECM** | **Epidermal proteins analyzed** | **Functional analyses** | **Reference (s)** |
| --- | --- | --- | --- | --- | --- | --- |
| 1. **Embryonic stem cells (ESCs)** | | | | | | |
| H9, I3, I6 | DMEM/Ham's F12 1:1 or FAD | BMP4 | PA6 cells fixed with 4% Formaldehyde | p63, K18, K14 | N. D. | (Aberdam et al., 2008) |
| H9 | FAD | BMP4 | MEF fixed with 4% Formaldehyde. Collagen I | K18, K12, Col IV | 3D Skin equivalents | (Hewitt et al., 2009) |
| H9, H13, H14; hiPSCs | DMEM/Ham's F12 1:1; D-KSFM | SU6656 (SFK: Src Family Kinase inhibitor); RA, BMP4 | MEF feeders; Matrigel | K8, K18, K14, K3; K10, IVL when Calcium was increased to 1mM |  | (Lian et al., 2013) |
| H1, H9 | D-KSFM | RA, BMP4 | Gelatin, Matrigel | p63, K18, K14; IVL, FLG, K10 | 3D Skin equivalents | (Metallo et al., 2008; Metallo et al., 2010) |
| H1 | a) FAD, D-KSFM; b) D-KSFM | a) AA, RA;  20 days later activin A b) RA | a) H1-derived Fibroblasts; Collagen IV b) Decellularized H1-derived Fibroblasts + Ficoll as crowding agent; Collagen IV | p63, K14 | N.D. | (Movahednia et al., 2015) |
| ESC KCL034; hiPSCs | mTESR1; FAD; D-KSFM; EpiLife + S7 supplement | RA, BMP4 | 3D decellularized Human dermal Fibroblasts; Collagen IV | p63, K14, Desmocollin 1 (DSC1), IVL, Filaggrin (FLG), Loricrin (LOR) | 3D skin equivalents | (Petrova et al., 2014) |
| hESCs; hiPSCs | StemXVivo Ectoderm kit | BMP4, DAPT, SB431542 |  | K18, K8, K19, p63, AP-2a, AP-2g | N.D. | (Qu et al., 2016) |
| H1 | DMEM/F12 (1:1) until D10; Thereafter D-KSFM for KC differentiation | BMP4, FCS +/-DAPT | Matrigel, Collagen I | K18, p63, K14 | Transgenic mice | (Tadeu and Horsley, 2013; Tadeu et al., 2015) |
| H9 | KSFM | RA, BMP4, AA | Matrigel | K14, p63 | tissue-engineered skin | (Zhao et al., 2020) |
| H1 and H9; iPSCs (ND1-4, NL-1, NL-4) | DMEM/F12 | SB431542 (D0-6), CHIR99021 (D1-5), BMP4 (D1-8), DAPT (D4-8), BMP4/DAPT/EGF (D9-11) | Vitronectin; albumin-free conditions | p63, K1, K10, K14 | KC transplanted to mouse excisional wound model | (Zhong et al., 2020) |
| 1. **Induced pluripotent stem cells (iPSCs)** | | | | | | |
| iPSCs | NOD Cg-Prkdc-scid IL2rgtm1Wjl/SzJ mice (NSG) |  | Teratoma | p63, K5, K10, IVL, FLG, Col VII, | Cells collected from teratoma. Skin equivalents grafted onto mice | (García et al., 2016) |
| hiPSCs | D-KSFM; KSFM | RA, BMP4 | Initially Matrigel. After passaging, Fibronectin and Collagen | p63, K14, DSG3, K1, COL7A1, Laminin 5, LOR | 3D Skin equivalents | (Itoh et al., 2011; Kajiwara et al., 2017; Matsumura et al., 2018) |
| hiPSCs | D-KSFM; CnT-07 after D4 | RA, BMP4 | Matrigel | H&E staining | 3D skin equivalent | (Itoh et al., 2013; Gledhill et al., 2015; Domingues et al., 2017; Matsumura et al., 2018) |
| hiPSCs | D-KSFM | RA+BMP4 until D4; hEGF, ROCKi | Vitronectin, Fibronectin + Collagen I | p63, K14, Laminin 5, K10, IVL, | 3D skin equivalents | (Kajiwara et al., 2017) |
| hiPSCs | DMEM:F12; D-KSFM; D-KSFM:KSFM | RA, BMP4, EGF | Collagen IV | p63, K5, K14 | 3D skin equivalents; Xenograft onto NOD/SCID mice | (Kim et al., 2018) |
| hiPSCs | hEB (after D4); D-KSFM (after D6) ; CNT-07 after D30 | RA, BMP4, ROCKi (until D10) | Collagen IV | p63, K14, ITGA6, ITGB4, DSC3, DSG3 | iPSC-derived KC with characteristics of basal epidermal KCs. | (Koch et al., 2022) |
| hiPSCs | D-KSFM; CnT-07 | RA, BMP4 | Geltrex + Collagen I => Col I + Col IV | K14 | - | (Kogut et al., 2014) |
| hiPSCs | D-KSFM | RA, BMP4 |  | K8, K18, p63, K14, | Skin equivalents | (Larribere et al., 2017) |
| hiPSCs | D-KSFM; CNT-07 (after D9) | RA and BMP4 (until D9); ROCKi [Y-27632] (after D28) | Matrigel; collagen (after D28) | p63, K14, K10, IVL, ITGB4, K5 | 3D skin equivalents | (Sah et al., 2021) |
| h 209.2 PSC | D-KSFM; CNT-07 after D4 | RA, BMP4 (until D4); ROCKi Y-27632 (after D10) | Fibronectin, Col IV | p63, ITGA6, INTβ4, COL17A1; also,　C1ORF68, LCE2B, TGM1, TGM2 | ESPCs exhibited basal cell phenotype | (Ruiz-Torres et al., 2021) |
| hiPSCs | FAD D4, N2 D7; D-KSFM | RA+ROCKi D2; RA+BMP4 D7; | Gelatin => MEF=> CELLstar | K18, p63, K14 | 3D skin equivalents; Mouse skin xenografts | (Sebastiano et al., 2014) |
| hiPSCs | FAD | BMP4, Ascorbic acid, TGF-b inhibitor (SB431542) | 3T3 feeders | p63, K14, K18 | - | (Shalom-Feuerstein et al., 2013) |
| hiPSCs | D-KSFM or CnT-07 until D22-28 =>CnT-57 | RA, BMP4 |  | DSG3, Col VII, Desmoplakin, K1, K5, K14, LOR | 3D skin equivalents In vivo reconstituted skin on SCID mice | (Umegaki-Arao et al., 2014) |
| hiPSCs | D-KSFM | RA, BMP4 | Collagen I | K18, TP63, K14 | HF morphogenesis in vivo in patch assay | (Veraitch et al., 2013) |
| hiPSCs | 1. FAD.  2. D-KSFM;  3. KSFM | Initially low BMP4, followed by RA, then RA+ BMP4+EGF, then BMP4+EGF, finally EGF alone | Matrigel. Differentiation on 3T3 feeders | CD200, ITGA6, K15, K5, K14, ITGB1, K1, K10 | Folliculogenic potential: 3D-skin equivalents; HF patch assay; Reconstitution assay (Si chamber). Permeability assays (toluidine blue dye) | (Yang et al., 2014a; Yang et al., 2014b) |
| 1. **Organoids** | | | | | | |
| hiPSCs | KCs: D-KSFM; Cnt-Prime Epithelial Medium \| Spheroid: D-KSFM; D-KFSM+EGF, BPE; D-KSFM+supplements; EBM; EGM | RA, BMP4 (until D6); KGF stabilized mRNA (D0 and D2) | - | K1, K10, K14, VIM | Organoids, xenotransplantation of skin wounds in mice | (Ebner-Peking et al., 2021) |
| hiPSCs | KCs => DMEM:F12 (D0-8); D-KFSM (D9-12); D-KFSM:KSFM (D13-30) \| Organoid => DMEM:F12 (3:1) (D5-7); DMEM:F12 (1:1) (D8-9); F12 (after D10) | RA, BMP4, EGF (until D12); BMP4, EGF (from D13-30) | Col IV (KCs); Col I (organoid) | p63, K5, K14, LOR | 3D skin organoid, engraftment in mice (cord blood mononuclear cells-derived iPSCs) | (Kim and Ju, 2019) |
| 1. **Patient-derived induced pluripotent stem cells** | | | | | | |
| hiPSCs (psoriasis patients) | DMEM/F12; DMEM/12+N2 (after D5); N2 (after D7) | RA, BMP4 (until D5); EGF (after D5) | Geltrex | p63, K1, K14, K18, LOR, IVL, LAM | KCs derived from PsO-iPSCs with dysregulated transcripts associated with psoriasis and KC differentiation | (Ali et al., 2020) |
| hiPSCs (Gorlin patients) | D-KSFM | RA, BMP4 (until D4); EGF (until 1^st^ passage); EGF, ROCKi [Y-27632] (after D4) | Matrigel (until D4); Col I, vitronectin, i-Matrix (after 1^st^ passage) | K14, ITGB4, p63 | N. D. | (Morita et al., 2021) |
| hiPSCs (RDEB patients) | E6; D-KSFM (after D7) | RA, BMP4 | Vitronectin | K14 | 3D skin equivalents; engraftment in mice | (Rami et al., 2020) |
| hiPSCs (ARCI1, ARCI2, TTD1, WT2) | KCM-mod | BMP4, RA (until D7) | 3T3 | p63, K5, K14, K18, IVL | basal keratinocyte-like cells derived from non-syndromic autosomal recessive congenital ichthyosis | (Lima Cunha et al., 2021) |
| hiPSCs (trisomy 21 rescued disomy 21) | D-KSFM | RA, BMP4; EGF (after D4); EGF + ROCKi [Y-27632] (after D14) | Vitronectin; Col I, fibronectin (after D14) | K10, K14, p63, IVL, LOR, FLG, ITGB4 | 3D skin equivalent | (Tanuma-Takahashi et al., 2021) |
| hiPSCs (RDEB patient-derived iPSCs) | E8; D-KSFM (after D7) | RA, BMP4 (until D7) | Vitronectin | K14, K10, LOR | 3D skin equivalents; engraftment in mice | (Jacków et al., 2019) |

**References**

Aberdam, E., Barak, E., Rouleau, M., De LaForest, S., Berrih-Aknin, S., Suter, D.M., et al. (2008). A pure population of ectodermal cells derived from human embryonic stem cells. *Stem cells* 26(2)**,** 440-444. doi: 10.1634/stemcells.2007-0588.

Ali, G., Elsayed, A.K., Nandakumar, M., Bashir, M., Younis, I., Abu Aqel, Y., et al. (2020). Keratinocytes derived from patient-specific induced pluripotent stem cells recapitulate the genetic signature of psoriasis disease. *Stem cells and development* 29(7)**,** 383-400. doi: 10.1089/scd.2019.0150.

Domingues, S., Masson, Y., Marteyn, A., Allouche, J., Perrier, A.L., Peschanski, M., et al. (2017). Differentiation of nonhuman primate pluripotent stem cells into functional keratinocytes. *Stem Cell Research & Therapy* 8(1)**,** 1-7. doi: 10.1186/s13287-017-0741-9.

Ebner-Peking, P., Krisch, L., Wolf, M., Hochmann, S., Hoog, A., Vári, B., et al. (2021). Self-assembly of differentiated progenitor cells facilitates spheroid human skin organoid formation and planar skin regeneration. *Theranostics* 11(17)**,** 8430. doi: 10.7150/thno.59661.

García, M., Quintana-Bustamante, O., Segovia, J.C., Bueren, J., Martinez-Santamaría, L., Guerrero-Aspizua, S., et al. (2016). Long-term skin regeneration in xenografts from iPSC teratoma-derived human keratinocytes. *Experimental Dermatology* 25(9)**,** 736-738. doi: 10.1111/exd.13049.

Gledhill, K., Guo, Z., Umegaki-Arao, N., Higgins, C.A., Itoh, M., and Christiano, A.M. (2015). Melanin transfer in human 3D skin equivalents generated exclusively from induced pluripotent stem cells. *PloS one* 10(8)**,** e0136713. doi: 10.1371/journal.pone.0136713.

Hewitt, K.J., Shamis, Y., Carlson, M.W., Aberdam, E., Aberdam, D., and Garlick, J.A. (2009). Three-dimensional epithelial tissues generated from human embryonic stem cells. *Tissue Engineering Part A* 15(11)**,** 3417-3426. doi: 10.1089/ten.tea.2009.0060.

Itoh, M., Kiuru, M., Cairo, M.S., and Christiano, A.M. (2011). Generation of keratinocytes from normal and recessive dystrophic epidermolysis bullosa-induced pluripotent stem cells. *Proc Natl Acad Sci U S A* 108(21)**,** 8797-8802. doi: 10.1073/pnas.1100332108.

Itoh, M., Umegaki-Arao, N., Guo, Z., Liu, L., Higgins, C.A., and Christiano, A.M. (2013). Generation of 3D skin equivalents fully reconstituted from human induced pluripotent stem cells (iPSCs). *PloS one* 8(10)**,** e77673. doi: 10.1371/journal.pone.0077673.

Jacków, J., Guo, Z., Hansen, C., Abaci, H.E., Doucet, Y.S., Shin, J.U., et al. (2019). CRISPR/Cas9-based targeted genome editing for correction of recessive dystrophic epidermolysis bullosa using iPS cells. *Proceedings of the National Academy of Sciences* 116(52)**,** 26846-26852.

Kajiwara, K., Tanemoto, T., Wada, S., Karibe, J., Ihara, N., Ikemoto, Y., et al. (2017). Fetal Therapy Model of Myelomeningocele with Three-Dimensional Skin Using Amniotic Fluid Cell-Derived Induced Pluripotent Stem Cells. *Stem Cell Reports* 8(6)**,** 1701-1713. doi: 10.1016/j.stemcr.2017.05.013.

Kim, Y., and Ju, J.H. (2019). Generation of 3D skin organoid from cord blood-derived induced pluripotent stem cells. *JoVE (Journal of Visualized Experiments)* (146)**,** e59297. doi: 10.3791/59297.

Kim, Y., Park, N., Rim, Y.A., Nam, Y., Jung, H., Lee, K., et al. (2018). Establishment of a complex skin structure via layered co-culture of keratinocytes and fibroblasts derived from induced pluripotent stem cells. *Stem Cell Research & Therapy* 9(1)**,** 1-10. doi: 10.1186/s13287-018-0958-2.

Koch, P.J., Webb, S., Gugger, J.A., Salois, M.N., and Koster, M.I. (2022). Differentiation of Human Induced Pluripotent Stem Cells into Keratinocytes. *Current Protocols* 2(4)**,** e408. doi: 10.1002/cpz1.408.

Kogut, I., Roop, D.R., and Bilousova, G. (2014). Differentiation of human induced pluripotent stem cells into a keratinocyte lineage. *Methods in Molecular Biology* 1195**,** 1-12. doi: 10.1007/7651_2013_64.

Larribere, L., Galach, M., Novak, D., Arevalo, K., Volz, H.C., Stark, H.J., et al. (2017). An RNAi Screen Reveals an Essential Role for HIPK4 in Human Skin Epithelial Differentiation from iPSCs. *Stem Cell Reports* 9(4)**,** 1234-1245. doi: 10.1016/j.stemcr.2017.08.023.

Lian, X., Selekman, J., Bao, X., Hsiao, C., Zhu, K., and Palecek, S.P. (2013). A small molecule inhibitor of SRC family kinases promotes simple epithelial differentiation of human pluripotent stem cells. *PLoS One* 8(3)**,** e60016. doi: 10.1371/journal.pone.0060016.

Lima Cunha, D., Oram, A., Gruber, R., Plank, R., Lingenhel, A., Gupta, M.K., et al. (2021). hiPSC-derived epidermal keratinocytes from ichthyosis patients show altered expression of cornification markers. *International Journal of Molecular Sciences* 22(4)**,** 1785.

Matsumura, W., Fujita, Y., Nakayama, C., Shinkuma, S., Suzuki, S., Nomura, T., et al. (2018). Establishment of integration-free induced pluripotent stem cells from human recessive dystrophic epidermolysis bullosa keratinocytes. *Journal of Dermatological Science* 89(3)**,** 263-271. doi: 10.1016/j.jdermsci.2017.11.017.

Metallo, C.M., Azarin, S.M., Moses, L.E., Ji, L., de Pablo, J.J., and Palecek, S.P. (2010). Human embryonic stem cell-derived keratinocytes exhibit an epidermal transcription program and undergo epithelial morphogenesis in engineered tissue constructs. *Tissue Eng Part A* 16(1)**,** 213-223. doi: 10.1089/ten.TEA.2009.0325.

Metallo, C.M., Ji, L., de Pablo, J.J., and Palecek, S.P. (2008). Retinoic acid and bone morphogenetic protein signaling synergize to efficiently direct epithelial differentiation of human embryonic stem cells. *Stem Cells* 26(2)**,** 372-380. doi: 10.1634/stemcells.2007-0501.

Morita, N., Onodera, S., Nakamura, Y., Nakamura, T., Takahashi, S.-i., Nomura, T., et al. (2021). Keratinocytes from Gorlin Syndrome-induced pluripotent stem cells are resistant against UV radiation. *Medical Molecular Morphology* 54(2)**,** 69-78.

Movahednia, M.M., Kidwai, F.K., Zou, Y., Tong, H.J., Liu, X., Islam, I., et al. (2015). Differential effects of the extracellular microenvironment on human embryonic stem cell differentiation into keratinocytes and their subsequent replicative life span. *Tissue Engineering. Part A* 21(7-8)**,** 1432-1443. doi: 10.1089/ten.TEA.2014.0551.

Petrova, A., Celli, A., Jacquet, L., Dafou, D., Crumrine, D., Hupe, M., et al. (2014). 3D in vitro model of a functional epidermal permeability barrier from human embryonic stem cells and induced pluripotent stem cells. *Stem Cell Reports* 2(5)**,** 675-689. doi: 10.1016/j.stemcr.2014.03.009.

Qu, Y., Zhou, B., Yang, W., Han, B., Yu-Rice, Y., Gao, B., et al. (2016). Transcriptome and proteome characterization of surface ectoderm cells differentiated from human iPSCs. *Scientific reports* 6(1)**,** 1-14. doi: 10.1038/srep32007.

Rami, A., Laczmański, L., Jacków-Nowicka, J., and Jacków, J. (2020). Reprogramming and Differentiation of Cutaneous Squamous Cell Carcinoma Cells in Recessive Dystrophic Epidermolysis Bullosa. *International Journal of Molecular Sciences* 22(1)**,** 245.

Ruiz-Torres, S., Lambert, P.F., Wikenheiser-Brokamp, K.A., and Wells, S.I. (2021). Directed differentiation of human pluripotent stem cells into epidermal stem and progenitor cells. *Molecular Biology Reports* 48(8)**,** 6213-6222. doi: 10.1007/s11033-021-06588-3.

Sah, S.K., Kanaujiya, J.K., Chen, I.-P., and Reichenberger, E.J. (2021). Generation of Keratinocytes from Human Induced Pluripotent Stem Cells Under Defined Culture Conditions. *Cellular Reprogramming* 23(1)**,** 1-13. doi: 10.1089/cell.2020.0046.

Sebastiano, V., Zhen, H.H., Haddad, B., Bashkirova, E., Melo, S.P., Wang, P., et al. (2014). Human COL7A1-corrected induced pluripotent stem cells for the treatment of recessive dystrophic epidermolysis bullosa. *Science Translational Medicine* 6(264)**,** 264ra163-264ra163. doi: 10.1126/scitranslmed.3009540.

Shalom-Feuerstein, R., Serror, L., Aberdam, E., Müller, F.-J., van Bokhoven, H., Wiman, K.G., et al. (2013). Impaired epithelial differentiation of induced pluripotent stem cells from ectodermal dysplasia-related patients is rescued by the small compound APR-246/PRIMA-1MET. *Proceedings of the National Academy of Sciences* 110(6)**,** 2152-2156. doi: 10.1073/pnas.1201753109.

Tadeu, A.M., and Horsley, V. (2013). Notch signaling represses p63 expression in the developing surface ectoderm. *Development* 140(18)**,** 3777-3786. doi: 10.1242/dev.093948.

Tadeu, A.M., Lin, S., Hou, L., Chung, L., Zhong, M., Zhao, H., et al. (2015). Transcriptional profiling of ectoderm specification to keratinocyte fate in human embryonic stem cells. *PLoS One* 10(4)**,** e0122493. doi: 10.1371/journal.pone.0122493.

Tanuma-Takahashi, A., Inoue, M., Kajiwara, K., Takagi, R., Yamaguchi, A., Samura, O., et al. (2021). Restoration of keratinocytic phenotypes in autonomous trisomy-rescued cells. *Stem Cell Research & Therapy* 12(1)**,** 1-11. doi: 10.1186/s13287-021-02448-w.

Umegaki-Arao, N., Pasmooij, A.M., Itoh, M., Cerise, J.E., Guo, Z., Levy, B., et al. (2014). Induced pluripotent stem cells from human revertant keratinocytes for the treatment of epidermolysis bullosa. *Science Translational Medicine* 6(264)**,** 264ra164-264ra164. doi: 10.1126/scitranslmed.3009342.

Veraitch, O., Kobayashi, T., Imaizumi, Y., Akamatsu, W., Sasaki, T., Yamanaka, S., et al. (2013). Human Induced Pluripotent Stem Cell–Derived Ectodermal Precursor Cells Contribute to Hair Follicle Morphogenesis In Vivo. *Journal of Investigative Dermatology* 133(6)**,** 1479-1488. doi: 10.1038/jid.2013.7.

Yang, R., Zheng, Y., Burrows, M., Liu, S., Wei, Z., Nace, A., et al. (2014a). Generation of folliculogenic human epithelial stem cells from induced pluripotent stem cells. *Nature communications* 5(1)**,** 1-11. doi: 10.1038/ncomms4071.

Yang, R., Zheng, Y., Li, L., Liu, S., Burrows, M., Wei, Z., et al. (2014b). Direct conversion of mouse and human fibroblasts to functional melanocytes by defined factors. *Nature Communications* 5**,** 5807. doi: 10.1038/ncomms6807.

Zhao, A., Yang, Y., Pan, X., Pan, Y., and Cai, S. (2020). Generation of keratinocyte stem‐like cells from human fibroblasts via a direct reprogramming approach. *Biotechnology Progress* 36(3)**,** e2961.

Zhong, H., Ren, Z., Wang, X., Miao, K., Ni, W., Meng, Y., et al. (2020). Stagewise keratinocyte differentiation from human embryonic stem cells by defined signal transduction modulators. *International Journal of Biological Sciences* 16(8)**,** 1450. doi: 10.7150/ijbs.44414.
