# Supplementary material for: Differentiation of pluripotent stem cells for modeling human skin development and potential applications
Source: Front Cell Dev Biol. 2022 Nov 23;10:1030339. doi: 10.3389/fcell.2022.1030339 (PMC9728031; doi:10.3389/fcell.2022.1030339)
Supplement: Supplementary file 2 [file Table2.docx]

**Supplementary Table 2**

Models of skin disorders using differentiation of human iPSCs.

| **Cell of origin** | **Disease** | **Reprogramming approach** | **Cell outcome** | **Proteins expressed** | **Research outcome** | **Reference (s)** |
| --- | --- | --- | --- | --- | --- | --- |
| Patient-derived human iPSCs from peripheral blood mononuclear cells | Psoriasis (PsO) | Sendai virus transduction | KCs^a^ derived from PsO-iPSCs | P63, K1, K14, K18, K19, LOR, IVL, LAM | These findings suggest that the pathogenesis of psoriasis is likely influenced by the genetic alterations of KCs. | (Ali et al., 2020) |
| Patient-derived human iPSCs from primary fibroblasts | Recessive dystrophic epidermolysis bullosa (RDEB) | Retroviral transduction | KCs derived from RDEB-iPSCs; 3D skin equivalents | p63, DSG3, K1, K5, K14, COL7A1, Laminin 5, Loricrin | Functional KC differentiation for effective iPSC-based therapy for epidermal diseases. | (Itoh et al., 2011) |
| Patient-derived human iPSCs from primary fibroblasts COL7A1 gene-corrected by CRISPR-Cas9 | Recessive dystrophic epidermolysis bullosa (RDEB) | Integration-free episomal vector | 3D human skin equivalents consisting of KCs and fibroblasts | K14, K10, loricrin | Demonstration of the feasibility of an iPSC-based gene correction strategy for the treatment of patients with RDEB (engraftment on mice) | (Jacków et al., 2019) |
| Patient-derived human iPSCs | Congenital ichthyosis | Lentiviral transduction | Basal KC-like cells | p63, K5, K14, K18, K10, IVL | Basal KC differentiation from human iPSCs established from ichthyosis patients | (Lima Cunha et al., 2021) |
| Patient-derived human iPSCs from peripheral blood mononuclear cells | Scleroderma (systemic fibrosis) | Sendai virus transduction | Skin organoid (dermal fibroblasts and KCs) | P63, K5, K14 | Drug screening of anti-fibrotic effect of raloxifene and bazedoxifene as potentially effective treatment of systemic sclerosis. | (Kim et al., 2022) |
| Human iPSCs | COVID-19 | - | Skin organoid (includes epidermis and dermis, cartilage and subcutaneous fat, and hair follicles) | P63, K14, K5, K10, K1, involucrin, ITGB1, DSG1, DSG2, laminin, COL4, nidogen, aggrecan, COL2A1 | SARS-CoV-2 can attack hair follicle and neurons in the skin. Potential platform for research on SARS-CoV-2 infection mechanisms and COVID-19 drug screening strategies (inflicted skin). | (Ma et al., 2022) |
| Patient-derived human iPSCs from oral fibroblasts | Gorlin syndrome (GS) | Sendai virus transduction | KCs derived from GS-iPSCs | K14, ITGB4, p63 | GS keratinocyte model. Resistance of GS KCs to apoptosis with a remarkable increase in BCL2 caused by UV, which may contribute to basal cell carcinomas. | (Morita et al., 2021) |
| Primary cells and human iPSCs from fibroblasts | Recessive dystrophic epidermolysis bullosa (RDEB) | Sendai virus transduction | Primary fibroblasts and fibroblast-derived MSCs^b^ (both fibroblasts COLJA1 gene-corrected with DNA cleavage followed by HDR applying an adenine base editor | - | Potential of adenine base editors to correct COLJA1 gene mutations as a promising strategy for autologous RDEB cell therapy. | (Osborn et al., 2020) |
| Patient-derived human iPSCs from primary dermal fibroblasts | Ectrodactyly, ectodermal dysplasia, and cleft lip/palate (EEC) syndrome | Lentiviral transduction | Mature KCs and corneal-epithelial development (two somatic mutations) | p63, K14, K18 | APR-246/PRIMA-1^MET,^ a small compound, reverted corneal epithelial lineage commitment and reinstated normal p63-related signaling pathway. | (Shalom-Feuerstein et al., 2013) |
| Patient-derived human iPSCs from primary fibroblasts  (Gene edited with CRISPR-Cas9 and TALENs) | Dominant dystrophic epidermolysis bullosa (DDEB) | Integration-free episomal vector | KCs and dermal fibroblasts | p63, K14, and COL7 (KCs) \| COL1, COL3, and COL7 (fibroblasts) | Knocking out of the COL7A1 mutant allele in DDEB via mutation site-specific mutagenesis NHEJ using CRISPR/Cas9 and TALENs. Differentiated KCs and fibroblasts could be potential sources of DDEB therapies. | (Shinkuma et al., 2016) |
| Patient-derived human iPSCs from amniotic fluid cells (trisomy 21 and rescued disomy 21) | Down syndrome | Integration-free episomal vector | KCs; 3D human skin equivalents (KCs + dermal fibroblasts) | K10, K14, p63, IVL, loricrin (expressed in trisomy rescued disomy 21) | Impairment in keratinocytic  differentiation in trisomy 21 iPSCs. In contrast, trisomy-rescued disomy 21 iPSC-derived KCs with an extended life capable of generating 3D skin with dermal fibroblast. | (Tanuma-Takahashi et al., 2021) |
| Patient-derived human iPSCs from fibroblasts  (Gene edited with CRISPR-Cas9, COL7A1) | Recessive dystrophic epidermolysis bullosa (RDEB) | Sendai virus transduction | KCs, MSCs, and haematopoietic cells | K5, K14, p63 (KCs) | Corrected fibroblasts of RDEB-causing mutations as iPSC sources for KC differentiation with potential application in cell therapies. | (Webber et al., 2016) |

^a^ KC: keratinocyte;  ^b^ MSC: mesenchymal stem cell

**References**

Ali, G., Elsayed, A.K., Nandakumar, M., Bashir, M., Younis, I., Abu Aqel, Y., et al. (2020). Keratinocytes derived from patient-specific induced pluripotent stem cells recapitulate the genetic signature of psoriasis disease. *Stem cells and development* 29(7)**,** 383-400. doi: 10.1089/scd.2019.0150.

Itoh, M., Kiuru, M., Cairo, M.S., and Christiano, A.M. (2011). Generation of keratinocytes from normal and recessive dystrophic epidermolysis bullosa-induced pluripotent stem cells. *Proceedings of the National Academy of Sciences of the United States of America* 108(21)**,** 8797-8802. doi: 10.1073/pnas.1100332108.

Jacków, J., Guo, Z., Hansen, C., Abaci, H.E., Doucet, Y.S., Shin, J.U., et al. (2019). CRISPR/Cas9-based targeted genome editing for correction of recessive dystrophic epidermolysis bullosa using iPS cells. *Proceedings of the National Academy of Sciences* 116(52)**,** 26846-26852.

Kim, Y., Nam, Y., Rim, Y.A., and Ju, J.H. (2022). Anti-fibrotic effect of a selective estrogen receptor modulator in systemic sclerosis. *Stem Cell Research & Therapy* 13(1)**,** 303. doi: 10.1186/s13287-022-02987-w.

Lima Cunha, D., Oram, A., Gruber, R., Plank, R., Lingenhel, A., Gupta, M.K., et al. (2021). hiPSC-derived epidermal keratinocytes from ichthyosis patients show altered expression of cornification markers. *International Journal of Molecular Sciences* 22(4)**,** 1785.

Ma, J., liu, J., Gao, D., Li, X., Zhang, Q., Lv, L., et al. (2022). Establishment of Human Pluripotent Stem Cell-Derived Skin Organoids Enabled Pathophysiological Model of SARS-CoV-2 Infection. *Advanced Science* 9(7)**,** 2104192. doi: 10.1002/advs.202104192.

Morita, N., Onodera, S., Nakamura, Y., Nakamura, T., Takahashi, S.-i., Nomura, T., et al. (2021). Keratinocytes from Gorlin Syndrome-induced pluripotent stem cells are resistant against UV radiation. *Medical Molecular Morphology* 54(2)**,** 69-78.

Osborn, M.J., Newby, G.A., McElroy, A.N., Knipping, F., Nielsen, S.C., Riddle, M.J., et al. (2020). Base Editor Correction of COL7A1 in Recessive Dystrophic Epidermolysis Bullosa Patient-Derived Fibroblasts and iPSCs. *Journal of Investigative Dermatology* 140(2)**,** 338-347.e335. doi: <https://doi.org/10.1016/j.jid.2019.07.701>.

Shalom-Feuerstein, R., Serror, L., Aberdam, E., Müller, F.-J., van Bokhoven, H., Wiman, K.G., et al. (2013). Impaired epithelial differentiation of induced pluripotent stem cells from ectodermal dysplasia-related patients is rescued by the small compound APR-246/PRIMA-1MET. *Proceedings of the National Academy of Sciences* 110(6)**,** 2152-2156. doi: 10.1073/pnas.1201753109.

Shinkuma, S., Guo, Z., and Christiano, A.M. (2016). Site-specific genome editing for correction of induced pluripotent stem cells derived from dominant dystrophic epidermolysis bullosa. *Proceedings of the National Academy of Sciences* 113(20)**,** 5676-5681. doi: doi:10.1073/pnas.1512028113.

Tanuma-Takahashi, A., Inoue, M., Kajiwara, K., Takagi, R., Yamaguchi, A., Samura, O., et al. (2021). Restoration of keratinocytic phenotypes in autonomous trisomy-rescued cells. *Stem Cell Research & Therapy* 12(1)**,** 1-11. doi: 10.1186/s13287-021-02448-w.

Webber, B.R., Osborn, M.J., McElroy, A.N., Twaroski, K., Lonetree, C.-l., DeFeo, A.P., et al. (2016). CRISPR/Cas9-based genetic correction for recessive dystrophic epidermolysis bullosa. *npj Regenerative Medicine* 1(1)**,** 16014. doi: 10.1038/npjregenmed.2016.14.
